# Associations of age at marriage and first pregnancy with maternal nutritional status in Nepal

**DOI:** 10.1093/emph/eoac025

**Published:** 2022-07-25

**Authors:** Jonathan C K Wells, Akanksha A Marphatia, Dharma S Manandhar, Mario Cortina-Borja, Alice M Reid, Naomi S Saville

**Affiliations:** Population, Policy and Practice Research and Teaching Department, UCL Great Ormond Street Institute of Child Health, London WC1N 1EH, UK; Department of Geography, University of Cambridge, Cambridge CB2 3EN, UK; Mother and Infant Research Activities, Kathmandu, Nepal; Population, Policy and Practice Research and Teaching Department, UCL Great Ormond Street Institute of Child Health, London WC1N 1EH, UK; Department of Geography, University of Cambridge, Cambridge CB2 3EN, UK; Institute for Global Health, University College London, London WC1N 1EH, UK

**Keywords:** child marriage, pregnancy, maternal nutrition, maternal capital, reproductive scheduling

## Abstract

**Background and objectives:**

Women’s nutritional status is important for their health and reproductive fitness. In a population where early marriage is common, we investigated how women’s nutritional status is associated with their age at marriage (marking a geographical transfer between households), and at first pregnancy.

**Methodology:**

We used data from a cluster-randomized control trial from lowland Nepal (*n* = 4071). Outcomes including body mass index (BMI) were measured in early pregnancy and trial endpoint, after delivery. We fitted mixed-effects linear and logistic regression models to estimate associations of age at marriage and age at pregnancy with outcomes, and with odds of chronic energy deficiency (CED, BMI <18.5 kg/m^2^), at both timepoints.

**Results:**

BMI in early pregnancy averaged 20.9 kg/m^2^, with CED prevalence of 12.5%. In 750 women measured twice, BMI declined 1.2 (95% confidence interval 1.1, 1.3) kg/m^2^ between early pregnancy and endpoint, when CED prevalence was 35.5%. Early pregnancy was associated in dose-response manner with poorer nutritional status. Early marriage was independently associated with poorer nutritional status among those pregnant ≤15 years, but with better nutritional status among those pregnant ≥19 years.

**Conclusions and implications:**

The primary determinant of nutritional status was age at pregnancy, but this association also varied by marriage age. Our results suggest that natal households may marry their daughters earlier if food insecure, but that their nutritional status can improve in the marital household if pregnancy is delayed. Marriage age therefore determines which household funds adolescent weight gain, with implications for Darwinian fitness of the members of both households.

## INTRODUCTION

The importance of mothers’ nutritional status for their offspring has been recognized by evolutionary biologists and public health nutritionists alike. When defining ‘parental investment’, for example, Trivers highlighted the importance of nutrition, further illustrated in his elucidation of ‘parent-offspring conflict’ over metabolic resources [1, 2]. Recently, the ‘developmental origins of adult health and disease’ (DOHaD) paradigm [3] has emphasized the penalties that arise when developing organisms are exposed to maternal undernutrition [4, 5]. In humans, for example, associations of birth weight and infant weight with adult health outcomes [4] demonstrate the importance of maternal nutritional investment during both pregnancy and lactation.

From an evolutionary perspective, life history theory assumes that females accumulate metabolic resources during the period of growth, in order to invest them in offspring under the selective pressure to maximize fitness [6]. Building on the concept of embodied capital [7], the term ‘maternal capital’ refers specifically to any component of maternal phenotype that promotes the capacity to invest in offspring [8], with nutritional status (metabolically active tissue and energy reserves in body fat) being a particularly important somatic component of maternal capital. Of relevance to both the DOHaD hypothesis and evolutionary public health, maternal capital not only enables nutritional investment but also represents the ‘ecological niche’ to which the foetus is exposed, indicating that any early developmental adjustments are directly related to maternal capital rather than to the external environment [8, 9].

In non-human species of mammal, the transition from growth to reproduction in females is regulated by endogenous factors (e.g. genotype, hormonal profile, social status) and exogenous factors (e.g. season, food supply) [6, 10, 11]. In human societies, however, a critical additional step is typically involved, namely the rite of passage of marriage. Our focus in this article is on traditional South Asian societies, where marriage is a powerful social norm that is near-universally followed and precedes pregnancy in the vast majority of women [12]. The study we describe below was carried out in such a society, in rural lowland Nepal.

Reproductive scheduling is an important component of variability in the capacity for maternal investment, and we argue that both social (marriage age) and physical (pregnancy age) markers of this scheduling merit attention. Beyond the implications for health, this issue is also of interest from an evolutionary perspective, because maternal nutritional status early in the reproductive career can potentially incorporate the imprint of two different households (natal and marital), which in turn may reflect contrasting opportunities of these households to maximize fitness through the medium of maternal capital.

### Public health perspective

Two widely used markers of maternal nutritional status in public health research are body mass index (BMI) and mid-upper arm circumference (MUAC). Higher maternal BMI and MUAC are protective against undernutrition in the offspring [13–17]. In India, for example, greater maternal BMI was associated with a lower risk of two markers of child undernutrition, stunting and wasting [18]. In turn, poor maternal nutritional status has been linked with stresses, such as poverty, lack of education and food insecurity [19, 20].

Of particular relevance here, adolescent reproduction has been associated with an increased risk of undernourished offspring [21], potentially because when the mother is still growing, she and the foetus may compete for nutrients [22, 23]. However, early women’s marriage has also been associated with an increased prevalence of child undernutrition [24, 25]. This association might broadly arise for several reasons. First, early marriage might simply be a marker of early reproduction. Second, marriage during adolescence may promote maternal psychosocial stress, which may then impair the transfer of nutrients to the foetus [26, 27]. In our study population, for example, we found that early marriage was associated with an increased prevalence of delivering the baby preterm [28]. Third, women married young may be especially low in the marital household hierarchy, in turn limiting their access to food. Due to cultural norms, in our study population, women typically eat last among all the household members [29]. Despite early marriage being linked with child undernutrition, however, very few studies have investigated its direct association with maternal nutritional status, and the results are heterogeneous across societies [25, 30, 31].

### Societal perspective

In South Asian societies, marriage typically has a transactional basis, where the decision for a girl to be married is made jointly by the natal and marital households. Such households often represent extended families, whereby a bride moves to live with her husband and his parents. Poverty in the natal household is widely considered to be a key driver of women’s early marriage [32, 33], but in our study population, it is low education rather than low household assets that predicts early marriage [34], and more broadly, a number of factors in both households may contribute [35, 36].

The timing of marriage can be approached through the lens of cost-benefit calculations, setting the unpaid work performed by the girl in each household against her demands on the food budget. On this basis, earlier marriage might be favoured by natal households experiencing food insecurity, allowing daughters to be offloaded from the household food budget. Moreover, though illegal, many families still pay dowries for their daughters [37]. Since older and more educated daughters require larger dowries [37, 38], this may also drive natal households with fewer resources and financial opportunities to marry their daughters earlier.

Marital households seek to gain new wives who will contribute not only labour but also children. A marital household might seek to recruit an older adolescent, whose accumulation of maternal capital (e.g. nutritional status, education) has already been funded by the natal household. However, they might also prefer a younger wife, as this might increase her compliance with the expectations of the existing household members, and she might also bear children sooner.

### Evolutionary perspective

From an evolutionary perspective, the nutritional status of women is a key determinant of fitness. However, the natal households and marital households have different opportunities to maximize inclusive fitness through this medium.

For the natal household, the opportunities to maximize fitness lie first in investing in the daughter during early life, to increase her maternal capital, and second by marrying her into a household able to provide further support for her reproductive career. As discussed above, whether natal households can marry their daughters into wealthier households with more resources, depending on their own resources and their ability to provide dowry. It is likely that food insecurity in the natal household will push the family to marry their daughter early, removing her demand on the family food budget. Broad support for this hypothesis comes from an intervention in India that promoted child nutrition in early life and was associated with greater schooling attainment and a reduced likelihood of daughters marrying early [39]. However, in part, because marriage decisions are often made at short notice, there are minimal data on women’s nutritional status immediately prior to marriage.

For the marital household, the fitness of its members can be maximized by drawing on both the new wife’s labour and her reproductive capacity. Young wives are typically under pressure to conceive soon after marriage [40, 41], and their nutritional status will affect the likelihood of this happening. Members of the marital household are genetically unrelated to the incoming wife, but (assuming her fidelity) will be related to the offspring that she produces [42]. Marital households can therefore maximize the fitness of their son either by recruiting a well-nourished wife, or by contributing their own resources to improve the wife’s nutritional status after marriage. Which of these alternative pathways occurs may be shaped by household economic circumstances and expectations, mediated by the incoming wife’s age at marriage.

**Figure 1.**
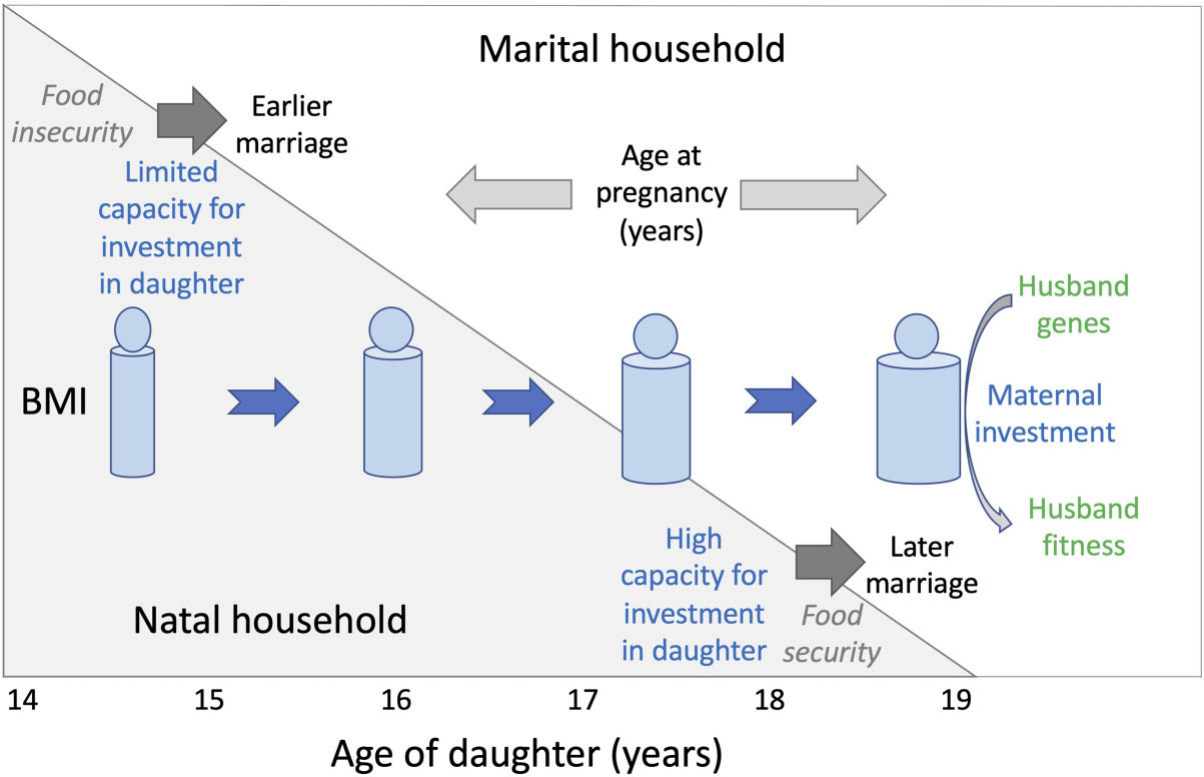
Conceptual diagram illustrating how the timing of the transition from the natal to the marital household may shape a woman’s nutritional status, as assessed by BMI. Natal households with lower resources and experiencing food insecurity may have limited capacity to invest in their daughter, which could lead to them marrying her at younger age, and with poor nutritional status. Natal households with greater resources may fund both their daughter’s education and her adolescent increase in BMI, leading to them marrying their daughter at a later age and with better nutritional status. These patterns interact with the daughter’s experience in the marital household, as they may shape both the timing of her pregnancy and her nutritional status when pregnancy occurs. Her nutritional status may continue to improve in the marital household if her pregnancy is delayed. These dynamics affect the fitness of the father, as maternal nutritional status represents a key developmental niche in which his genes are expressed in the offspring

Because nutritional status typically increases during adolescence, indicated by rising height, BMI and MUAC with age [43], the timing of both women’s marriage and pregnancy therefore has implications for the fitness of members of both households (Fig. 1). Specifically, in bio-economic terms, the timing of marriage determines the extent to which each household funds the adolescent accumulation of maternal capital; conversely, the timing of pregnancy determines when this store of capital is ‘cashed in’ and converted into the first bout of reproductive investment.

### Hypotheses

In lowland Nepal, where our study is based, the majority of women are married relatively early in adolescence and typically bear children within a few years. For example, in earlier analyses of our cohort, 85% of women had been married before 18 years [44], considered by national legislation and the UN as the minimum acceptable age [45]. Child undernutrition is common [46], but the level of undernutrition among mothers at the onset of the reproductive career is less well studied. In this context, we therefore aimed to investigate how both social and physiological markers of the reproductive career are associated with women’s nutritional status.

We tested two primary hypotheses, related to direct associations of age at pregnancy and marriage with our outcomes, markers of nutritional status:

H1: that earlier first pregnancy is associated with poorer nutritional status, in dose-response manner. This association is predicted on the basis that mothers reproducing earlier have had less time to accumulate somatic capital.

H2: that earlier marriage is associated with poorer nutritional status, in dose-response manner. This association is predicted on the basis that mothers married earlier may have faced greater exposure to adverse social factors (constraints on accessing adequate food in both natal and marital households, and psychosocial stress in the marital household).

We ran these analyses at two timepoints, to explore associations of marriage and pregnancy age with markers of nutritional status (i) during early pregnancy, before any substantial weight gain specific to reproduction had occurred, and (ii) after birth, when costs of pregnancy have been met and those of lactation are ongoing or completed. All analyses adjusted for household socioeconomic factors.

To our knowledge, no previous study has evaluated associations of both age at marriage and age at pregnancy with maternal nutritional status. We therefore aimed to disentangle these associations while controlling for potential confounders, by testing a third hypothesis:

H3: that the associations of marriage age and pregnancy age with nutritional status are independent of each other.

## METHODS

We analysed data from the cluster-randomized controlled Low Birth Weight South Asia Trial (LBWSAT) in rural lowland Nepal (Terai), which assessed the impact of interventions during pregnancy on birth weight and infant growth [47]. The trial spanned 80 geographic clusters (Village Development Committees, VDCs) in southern Mahottari and Dhanusha districts, bordering Bihar state in India. The Maithili-speaking Madhesi women of our study have the lowest median marriage age (15 years) and educational attainment (about three-quarters have no schooling) in Nepal [44, 48]. Families generally decide when and to whom girls are married [49].

Of ∼64 000 married women consenting to menstrual monitoring, 25 090 pregnant women aged 10–49 years were recruited into LBWSAT (December 2013 to February 2015). Interventions included: (i) Women’s Groups using the Participatory Learning and Action (PLA) behaviour change approach, (ii) PLA and unconditional cash transfers and (iii) PLA and food supplementation, compared with (iv) a control group accessing Government of Nepal health services [50]. The primary outcome of the trial was offspring birth weight.

The Nepal Health Research Council, University College London and University of Cambridge granted research ethics approvals for secondary analysis of LBWSAT data. VDC secretaries consented to the inclusion of clusters. Written consent was obtained from women and from guardians for married adolescents below age of 18 years.

### Data acquisition and preparation

Women’s age at marriage and first pregnancy was recorded in ‘running years’ converted to completed years (running years minus 1). This is because most people count age in the year they are running (e.g. their 18th year) rather than in completed years (e.g. they are 17.5 years old) in this setting. Marriage age was categorized into four groups (≤14, 15, 16 or ≥17 years), and pregnancy age into five groups (≤15, 16, 17, 18 or ≥19 years) reflecting the distribution of the data. According to the Nepali education system [51], education of both the woman and her husband was categorized into four groups: none, primary (1–5 years), lower secondary (6–8 years) or secondary/higher (9+ years). Caste was categorized into three groups: disadvantaged (Muslim or Dalit); Middle (Janjati, Terai castes); and Advantaged (Yadav, Brahmin).

A household asset score was constructed using principal component analysis from assessment of 11 material assets, including consumer goods (television, motorbike, computer) and infrastructure (drinking water source, toilet facilities, flooring, roof and wall materials, number of rooms, access to electricity, non-bio-mass fuel use). The first component had positive factor loadings for all 11 variables and was taken as the marker of wealth, accounting for 31.2% of the variability. The score was categorized in quartiles, with 1 the poorest. Separately, households were also categorized as holding land, or not.

For the assessment of nutritional status, BMI was calculated from measurements of weight (Tanita solar scales) and height (Shorr boards), and MUAC measured with a non-stretchable tape [50]. BMI and MUAC were measured at different timepoints in the trial, depending on when individual women attended clinics. We used data from ‘early pregnancy’ (8–13 weeks) or ‘trial endpoint’, which was assessed within 25 months after delivery. Chronic energy deficiency (CED) was categorized as BMI <18.5 kg/m^2^ [52].

Information on contraception use in the month prior to the pregnancy was available for a subsample. The data were obtained using the London Measure of Unplanned Pregnancy (LMUP) questionnaire, which has been validated in low-income settings such as India [53]. Contraception use was coded as a binary variable (yes/no).

### Sample selection

We restricted the analysis to first-time mothers, as the first pregnancy is assumed to be most strongly exposed to age at marriage. We excluded 877 mothers who lacked data for marriage age. We also excluded those married before 10 years (*n* = 35) or after 23 years (*n* = 34), as these ages were atypical of the population. Given the norm for first pregnancy during adolescence or early adulthood, we also excluded those whose first pregnancy occurred >30 years (*n* = 114).

### Statistical methods

We summarized women’s age at marriage and pregnancy with median and interquartile range (IQR). The anthropometric outcomes were tested for normality using the Shapiro–Wilks test. All four variables satisfied the criteria of normality and were analysed without transformation. We compared women with and without anthropometric data to assess bias.

We used linear regression analysis to describe unadjusted associations of marriage and pregnancy age with the outcomes. We then fitted mixed-effects models with a random effect on the intercept accounting for within-cluster variability that adjusted for potential confounders: caste, maternal and paternal education, asset score, landholding, trial arm and cluster. In these models, we adjusted pregnancy models for marriage age, and vice versa. We used dummy variables for specific categories of the following confounders: education, caste, trial arm, landholding, in each case with the highest value as the reference category. We analysed asset score as a continuous variable. For the endpoint data, we also adjusted for the time since the birth of the infant.

To test associations with CED, we used mixed-effects logistic regression models, again accounting for clusters and confounders. We estimated odds ratios (OR) of CED, using the same confounders as above. For these models, we analysed assets categorized in quartiles. The higher value of each trait (e.g. richest household) was set as the reference group for exposure variables.

We focus on coefficients of the magnitude of effect and their 95% confidence intervals (95% CI) and also provide *P*-values for information. All analyses were conducted in SPSS version 27 (IBM Corporation).

## Results

A flow chart describing numbers available for analysis is given in Supplementary Fig. S1. There were 7474 eligible first-time mothers; however, our analysis was restricted to 4071 women with at least one anthropometric measurement. These women represent a younger generation within the LBWSAT cohort, likely to be wealthier and more educated than multigravidae. There was no difference in age at pregnancy or anthropometric measurements between those with/without data on marriage age (*P* > 0.4 in all cases). Women lacking anthropometric data were younger at marriage (Δ = 0.1 years, 95% CI 0.0, 0.2) and older at pregnancy (Δ = 0.2 years, 95% CI 0.1, 0.3) than those with data, but the magnitudes of effect were trivial. The final sample comprised 1409 women for early pregnancy and 3412 women for endpoint.

**Figure 2.**
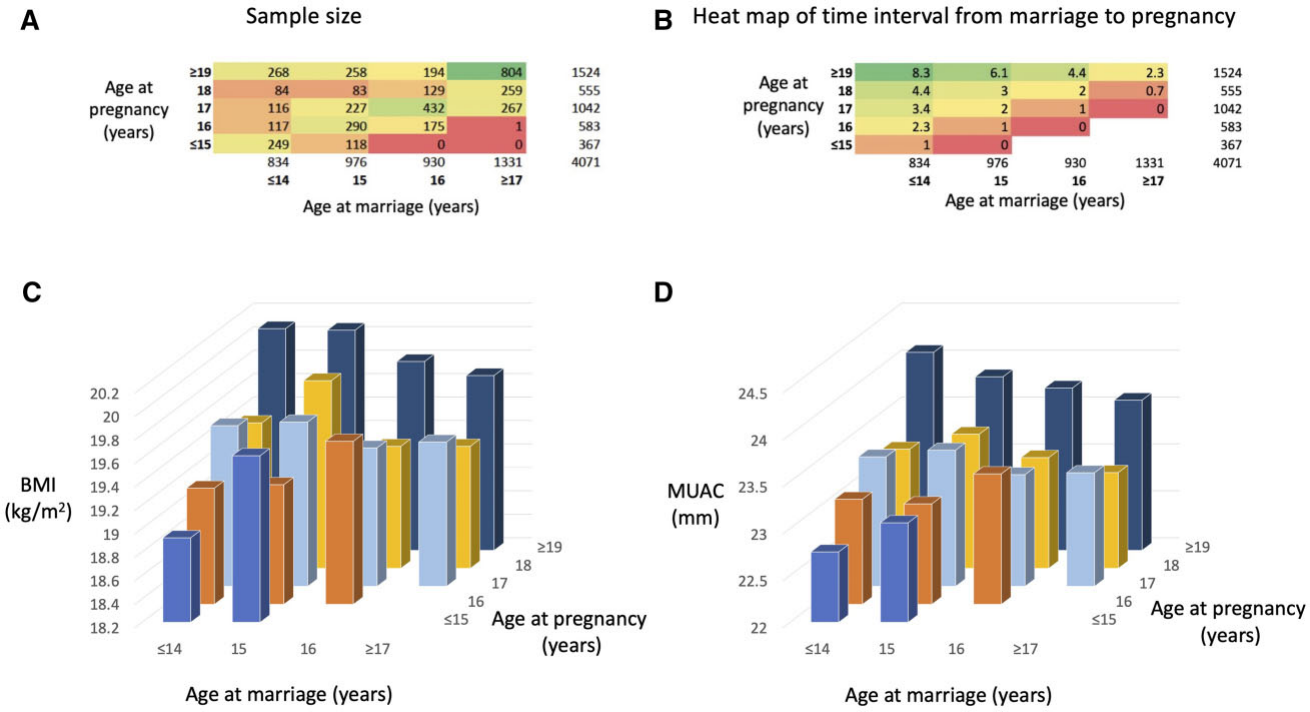
Interactive associations of nutritional status with age at marriage and age at first pregnancy. (**a**) Heat map of age at pregnancy and age at marriage for the sample of 4071 women. Twenty percent of the cohort had been married ≤14 years, and 49% had their first pregnancy ≤17 years. (**b**) Heat map of the time interval (years) between marriage and pregnancy. (**c**) Plot for BMI. (**d**) Plot for MUAC. Among those pregnant at ≤15 years, earlier marriage was associated with lower BMI and MUAC (*P* < 0.05). Among those pregnant at ≥19 years, however, earlier marriage was associated with higher BMI (*P* < 0.05 for trend)

Table 1 summarizes the characteristics of the sample. Almost half of the women (48%) and 40% of their husbands had never attended any school, and only 24% of women and 30% of men had attended secondary school. Almost one-third were from the disadvantaged castes. Over two-thirds of the women (68%) were from households that owned some land. Median age at marriage was 16 years IQR 2) and age at pregnancy was 18 years (IQR 2), with only 14% marrying at or after 18 years. A heat map summarizing the distributions of age at marriage and pregnancy is given in Fig. 2a.

**Table 1.** Summary statistics of the sample (*n *=* *4701)

	*n*	%	
Age at marriage (year)			
≤14	834	20.5	
15	976	24.0	
16	930	22.8	
≥17	1331	32.7	
Age at pregnancy (year)			
≤15	367	9.0	
16	583	14.3	
17	1042	25.6	
18	555	13.6	
≥19	1524	37.4	
Maternal education			
Never went to school	1950	47.9	
Primary	510	12.5	
Lower secondary	632	15.5	
Secondary or higher	979	24.0	
Husband’s education			
Never went to school	1638	40.2	
Primary	464	11.4	
Lower secondary	745	18.3	
Secondary or higher	1224	30.1	
Caste			
Dalit/Muslim disadvantaged	1303	32.0	
Janjati/middle class	1815	44.6	
Yadav/Brahmin least disadvantaged	953	23.4	
Land ownership^a^			
No	1295	31.8	
Yes	2775	68.2	
Asset quintile^a^			
1	767	18.8	
2	940	23.1	
3	1147	28.2	
4	1216	29.9	
Study arm			
Control	957	23.5	
Women’s group	914	22.5	
Cash	1215	29.8	
Food	985	24.2	
	n	Mean	SD
Height (cm)	4052	150.8	5.4
BMI (kg/m^2^)			
Early pregnancy	1409	20.9	2.2
Endpoint	3385	19.6	2.4
MUAC (cm)	n	Mean	SD
Early pregnancy	1409	23.5	2.0
Endpoint	3412	23.4	2.2

a Missing data (*n *=* *1).

During early pregnancy, mean BMI was 20.9 kg/m^2^. In 750 women with data at both timepoints, BMI declined by 1.2 (95% CI 1.0, 1.3) kg/m^2^ between early pregnancy and endpoint, on average assessed at 9 (SD 5) months after delivery. This decline indicates that reproduction imposed significant energetic costs. In the complete sample, the prevalence of CED rose from 12.3% in early pregnancy to 35.5% at endpoint. However, there was negligible longitudinal change in MUAC in this subsample (Δ = 0.0 cm, 95% CI −0.1, 0.1).

In crude linear regression models, earlier age at marriage was not associated with BMI or MUAC during early pregnancy. However, earlier pregnancy was associated with lower values for both outcomes, with indications of an inverse dose-response association for BMI (Table 2). The results were similar when the models were controlled for caste, maternal and paternal education, household assets and land ownership, study arm and cluster, and marriage age.

**Table 2.** Associations of age at marriage and pregnancy with BMI and MUAC in early pregnancy and endpoint

	Early pregnancy (*n *=* *1409)						Endpoint (*n *=* *3412)^a^					
	BMI (kg/m^2^)			MUAC (cm)			BMI (kg/m^2^)			MUAC (cm)		
	*B*	95% CI	*P*-value	*B*	95% CI	*P*-value	*B*	95% CI	*P*-value	*B*	95% CI	*P*-value
Marriage age												
Crude model (years)												
≤14	−0.22	−0.56, 0.12	0.2	−0.04	−0.35, 0.26	0.7	−0.04	−0.26, 0.19	0.7	−0.01	−0.21, 0.20	0.9
15	−0.06	−0.38, 0.26	0.7	−0.04	−0.32, 0.24	0.7	0.08	−0.14, 0.29	0.4	0.01	−0.19, 0.21	0.9
16 y	−0.25	−0.55, 0.05	0.1	−0.06	−0.33, 0.21	0.6	−0.08	−0.30, 0.14	0.4	−0.08	−0.29, 0.12	0.4
17+	Ref			Ref			Ref			Ref		
Adjusted model^b^ (year)												
≤14	0.07	−0.32, 0.46	0.7	0.08	−0.26, 0.43	0.4	0.33	0.08, 0.58	0.009	0.42	0.19, 0.65	<0.001
15	0.28	−0.08, 0.65	0.1	0.07	—0.25, 0.39	0.2	0.43	0.0.19, 0.66	<0.001	0.35	0.14, 0.57	0.001
16	−0.00	—0.32, 0.33	0.9	0.06	−0.23, 0.35	0.6	0.21	−0.02, 0.45	0.078	0.21	0.00, 0.43	0.049
17+	Ref			Ref			Ref			Ref		
Pregnancy age												
Crude model (years)												
≤15	−0.98	−1.42, −0.54	<0.001	−0.62	−1.01, −0.22	0.002	−0.87	−1.17, −0.57	<0.001	−0.99	−1.27, −0.71	<0.001
16	−0.72	−1.08, −0.37	<0.001	−0.30	−0.62, 0.01	0.057	−0.55	−0.79, 0.30	<0.001	−0.60	−0.83, −0.37	<0.001
17	−0.47	−0.77, −0.17	0.002	−0.41	−0.68, −0.15	0.002	−0.45	−0.65, −0.24	<0.001	−0.51	−0.70, −0.32	<0.001
18	−0.52	−0.89, −0.14	0.007	−0.52	−0.86, −0.19	0.002	−0.61	−0.85, −0.36	<0.001	−0.66	−0.89, −0.43	<0.001
19+	Ref			Ref			Ref			Ref		
Adjusted model^b^ (year)												
≤15	−1.05	−1.55, −0.26	<0.001	−0.61	−1.04, −0.16	0.007	−0.88	−1.21, −0.56	<0.001	−1.08	−1.38, −0.78	<0.001
16	−0.80	−1.20, −0.41	<0.001	−0.32	−0.67, 0.03	0.078	−0.69	−0.96, −0.42	<0.001	−0.70	−0.95, −0.46	<0.001
17	−0.43	−0.74, −0.11	0.008	−0.38	−0.66, −0.10	0.008	−0.46	−0.68, −0.24	<0.001	−0.52	−0.72, −0.33	<0.001
18	−0.42	−0.80, −0.05	0.028	−0.46	−0.79 −0.13	0.007	−0.47	−0.72, −0.22	<0.001	−0.58	−0.80, −0.35	<0.001
19+	Ref			Ref			Ref			Ref		

a Twenty-six missing data points for BMI.

b Adjusted models are mixed-effects models, controlling for caste, maternal and paternal education, land ownership, assets, cluster and study arm. In addition, marriage age model controlled for pregnancy age, and vice versa.

Likewise, earlier age at marriage was not associated with BMI or MUAC at endpoint. However, in adjusted models, the associations became positive and significant (Table 2). For example, marriage at 14 years or below was associated with 0.3 (95% CI 0.1, 0.6) kg/m^2^ greater BMI, and 0.4 (95% CI 0.2, 0.6) cm greater MUAC, and similar though smaller effects were observed for marriage at 15 and 16 years. In contrast, associations of early pregnancy with BMI and MUAC at endpoint remained negative and showed broadly dose-response associations. For example, in adjusted models, pregnancy at ≤15 years was associated with −0.9 (95% CI −1.2, −0.6) kg/m^2^ lower BMI, and −1.1 (95% CI −1.4, −0.8) cm lower MUAC.

These contrasting associations in adjusted models suggest that once the negative association of early pregnancy is taken into account, earlier marriage is associated with higher BMI and MUAC. However, the pattern is complex and shows an interaction between the two exposures (Fig. 2). This arises because the relationship between marriage age and pregnancy age is a function of the time spent in the marital household prior to becoming pregnant (Fig. 2b). For women pregnant ≤15 years, those married at ≤14 years had 0.8 (95% CI 1.5, 0.2) kg/m^2^ lower BMI and 0.6 (1.2, 0.1) cm lower MUAC at endpoint, compared to those married at 15 years. Conversely, among those pregnant ≥19 years, BMI was 0.5 (95% CI 0.1, 0.9) kg/m^2^ higher for those married at ≤14 years, and 0.5 (95% CI 0.1, 0.9) kg/m^2^ higher for those married at 15 years, compared to those married at ≥17 years. An interaction term for pregnancy × marriage age was significant in the BMI model (*P* = 0.027). There were similar increments of 0.6 (95% CI 0.2, 0.9) cm and 0.43 (0.0, 0.8) cm greater MUAC among those married at ≤14 and 15 years respectively, compared to those married at ≥17 years, with again evidence of an interaction for pregnancy × marriage age (*P* = 0.06).

The continuous associations of marriage and pregnancy with BMI and MUAC were broadly replicated regarding CED risk, analysed using mixed-effects logistic regression models. In early pregnancy, younger age at pregnancy but not early marriage was associated with greater risk (Table 3). The risk was elevated among those pregnant at ≤15 years (OR 2.1, 95% CI 1.1, 4.2) with weaker associations also evident for those pregnant at 16 and 18 years. At endpoint, earlier pregnancy likewise showed elevated risks, again most evident in those pregnant ≤15 years (OR 1.6, 95% CI 1.2, 2.1). However, at this timepoint, early marriage independently showed protective effects, evident for both those marrying ≤14 and 15 years (OR 0.8, 95% CI 0.6, 1.0 in both cases).

**Table 3.** Associations of age at marriage and pregnancy with the risk of CED in early pregnancy and at endpoint

	Early pregnancy (*n *=* *1409)			Endpoint (*n *=* *3385)		
	OR	95% CI	*P*-value	OR	95% CI	*P*-value
Marriage age						
≤14	0.93	0.53, 1.64	0.8	0.76	0.61, 0.96	0.019
15	0.83	0.49, 1.41	0.4	0.76	0.62, 0.95	0.013
16	1.08	0.69, 1.71	0.7	0.90	0.73, 1.11	0.3
17+	1.00			1.00		
Pregnancy age						
≤15	2.13	1.09, 4.16	0.089	1.57	1.17, 2.10	0.003
16	1.59	0.90, 2.79	0.1	1.24	0.97, 1.58	0.083
17	1.27	0.80, 2.01	0.3	1.11	0.91, 1.35	0.2
18	1.58	0.94, 2.66	0.085	1.21	0.97, 1.51	0.094
19+ year (ref)	1.00			1.00		

Mixed-effects logistic regression models adjusting for caste, maternal and paternal education, land ownership, asset quartile, study arm and cluster.

CED categorized as BMI <18.5 kg/m^2^.

Data on contraception use in the month prior to the pregnancy were available for a subsample of 2823 women, representing 69% of the total sample. The overall rate was 16.5%. Variability in the use of contraception by marriage age and pregnancy age is given in Supplementary Fig. S3. Among those married ≤15 years, the rate of contraception use was higher among those pregnant later (e.g. <16 years: 9.8% vs 18 years: 26.3%), except for those pregnant at 19+ years (12.4%), with weaker patterns evident for other marriage ages. Among those pregnant at 18+ years, there was no difference in BMI in early pregnancy among those using contraception in the last month, compared to those not (Δ = 0.1 kg/m^2^, 95% CI −0.6, 0.7).

## DISCUSSION

We aimed to disentangle associations of women’s age at marriage and pregnancy with markers of their nutritional status, among a population of first-time mothers in a low-income setting in lowland Nepal. In this population, the decision for girls to be married is made not by themselves, but by other members of their household along with the marital household. If the schedule of marriage and pregnancy influences women’s nutritional status, this therefore represents the impact on women of broader ‘societal transactions’ over household subsistence and reproduction, which from an evolutionary perspective are relevant to survival and fitness [54]. The timing of marriage then shapes the impact of the two households. While studies of young women’s nutritional status typically focus on the implications for the health of the next generation, through the lens of the DOHaD hypothesis, we emphasize the importance of this issue for the health and well-being of the women themselves. This is particularly the case in the Maithili-speaking population we studied, where young brides are relatively isolated after marriage and are considered very low in the marital household hierarchy [49, 55]. Although we have previously shown that women’s education is the primary factor associated with later marriage in this population, education may not lead to women’s autonomy, and there is strong pressure to bear children soon after marriage. Amongst those married later, the time to the first pregnancy was faster [44], which may indicate both better physiological ability to conceive and pressure to conceive.

Overall, the population demonstrates substantial exposure to energy stress, with 12.5% of the sample categorized as having CED in early pregnancy. While this may in part reflect a generic tendency for slim physique in south Asian population that appears to have been present for millennia [56], the average decline of 1.1 kg/m^2^ in BMI over a single reproductive event confirms the energetic stress associated with reproduction, while food insecurity may also be a challenge. The overall loss of BMI, combined with the negative slope of BMI with time after delivery, indicates that lactation is the primary cause of these costs, consistent with other data [57, 58]. After the first pregnancy, the prevalence of CED had risen to 35.5%.

Beyond these overall patterns, we found that nutritional status varied systematically in association with the timing of both pregnancy and marriage, moreover with an interactive association between these two variables. Our main findings supported H1, showing that early pregnancy was the primary marker of lower BMI and MUAC. Both in early pregnancy and trial endpoint, we identified a dose-response association, whereby the earlier the pregnancy, the lower the BMI and MUAC values. The magnitude of effect was substantial. For example, among those marrying ≤15 years, the ∼1.1 kg/m^2^ deficit in BMI in those pregnant by 15 years, compared to those pregnant at 19+ years, is equivalent to 2.5 kg of weight for a woman of average height in this population. If this weight entirely comprised fat, it would contain ∼99 MJ of energy reserves [59], which would likely benefit reproductive investment throughout the woman’s reproductive career. Pregnancy before 17 years was also associated with an increased risk of CED at both early and late pregnancy. These analyses show that women who reproduce at young age may pay fitness penalties, being more likely to produce offspring prone to undernutrition and early mortality compared to better-nourished mothers, despite an early start to their reproductive career.

Conversely, results for H2, testing associations of marriage age with nutritional status, were largely null, except at trial endpoint where the adjusted associations were the reverse of what we predicted. We observed higher BMI and MUAC among those marrying earlier, but only in models that adjusted for pregnancy age. In adjusted models, earlier marriage also showed a trend towards lower risk of CED. Interpretation of these unexpected findings is therefore aided by our interaction analyses, exploring in more detail the independent associations of marriage and pregnancy age. However, as we did not hypothesize in advance what pattern these analyses might show, we present these data as hypothesis-generating.

### Hypothesis-generating results

Our interaction analyses showed that the association of marriage age with nutritional status varied, depending on pregnancy age. Among women who became pregnant very early (≤15 years), we found that those who were also married early had significantly lower BMI at endpoint compared to those who were married later. We suggest that for women who reproduce early, being married at young age is associated with thinness, which may indicate that food insecurity in the natal household is one of the factors contributing to the decision for early marriage. An important methodological point is that, due to the nature of the study design, the women who were pregnant at ≤15 years had their measurements of nutritional status relatively soon after marriage (Supplementary Fig. S2), increasing reliability of the association. We therefore consider the alternative explanation that the early-marrying group lost more weight after being married, less likely. Overall in this population, BMI increases with maternal age, especially during adolescence [60].

At the other extreme, among those who became pregnant relatively late (≥19 years), earlier marriage was associated with higher BMI. This finding was supported by equivalent analysis of CED risk, which found that while early pregnancy was associated with elevated risk, earlier marriage was associated with reduced risk. There are two possible explanations for this finding. Arguably the most likely explanation is that even if girls who are married young are relatively thin (as discussed above), they are able to improve their nutritional status if they do not reproduce soon after marriage. The longer the time interval in the marital household between marriage and reproduction, the better the woman’s nutritional status when she does reproduce.

There is an alternative explanation, which we cannot rule out due to the lack of data on nutritional status at the time of marriage. Potentially, some women were selected for early marriage specifically because they had higher BMI, and these women were then also able to delay childbearing, resulting in correlations of early marriage, higher BMI and relatively late childbearing. In a prospective cohort study in India, for example, we found that girls who were married early gained BMI faster during adolescence than their unmarried peers [61]. This BMI gain might indicate a social signal of ‘readiness for marriage’, even though in this case it was unrelated to age at menarche. This explanation might also apply to our study population, but we suggest it may be less important. It is not consistent with the association of BMI with early marriage among those whose nutritional status was measured much closer in time to marriage.

Although not a natural fertility population, access to contraception in our sample appeared limited to a minority. In the subsample of women for whom data on contraception in the month prior to pregnancy were available, rates of contraception were lower amongst early-marrying women if they became pregnant earlier (<16 years) compared to later (18 years). Therefore, the use of contraception is one pathway through which later age at pregnancy and higher BMI may be related in this population, though there was no evidence of a direct link between recent contraception use and BMI. Notably, contraception rates fell again among those pregnant at 19+ years, indicating that eventually, the desire for children supersedes prevention.

Overall, these interaction analyses offer hypotheses that may stimulate further work, investigating how women’s nutritional status may be influenced by decisions of both natal and marital households over the timing of women’s marriage. However, obtaining prospective data on this issue, in this setting, may be very difficult.

Our findings for BMI and MUAC contrast with those reported previously for height in this cohort [62]. Among women aged 23+ years, assumed to have completed their linear growth, earlier marriage and earlier first pregnancy were both independently associated with shorter height, indicating costs of reproductive scheduling that at a mechanistic level might involve both the stress response (early marriage) and competition for nutrients between mother and foetus (early pregnancy) [22]. Collectively, therefore, our analyses suggest that following early marriage, women can recover BMI but not height if they delay pregnancy.

### Broader interpretation

These patterns are of particular interest in the context of debates over the drivers of early marriage. It is widely assumed that poverty in the natal household is the primary driver of early marriage [32, 33]. However, we did not observe this association, rather the primary predictor of early marriage in the natal household was lack of education [34]. We also found that early marriage was associated with greater land ownership of the marital household, suggesting that natal households were willing to marry their daughters early in order to access this form of geographic capital [63].

Our findings suggest that food insecurity may be another factor driving early marriage and that families may marry their daughters at younger age if they are struggling to feed them adequately. This is consistent with the association of early marriage with low BMI among those measured soon after marriage. If we accept the more likely hypothesis that women gain in BMI after marriage and have the highest BMI if they marry young and become pregnant later, then both the natal and marital families can shape the nutritional status, and hence the reproductive fitness, of the daughter through decisions about marriage age and pregnancy age. Natal families may give their daughter better nutritional circumstances if they marry them earlier, but only if the marital household has food security. Further improvements in young brides’ nutritional status might occur if childbearing was delayed, which might occur through deliberate efforts (contraception) or through physiological pathways.

In our study, the relative importance of biological factors (influencing reproductive energetics) versus active decision-making (e.g. contraception) in shaping the schedule of pregnancy after marriage remains unclear. Of women who married before 15 years, 44% were already pregnant by 15 years, indicating no universal constraint of age on fertility; nonetheless 32% only became pregnant at 19+ years. Our analyses suggest that access to contraception contributes to this variability, but we were only able to analyse contraception behaviour in the month prior to the pregnancy and hence do not know the full magnitude of effect. The trend towards increasing BMI over adolescence in this population [60] may contribute to greater physiological capacity to conceive with age; however, other factors such as reproductive maturity may also be important. The normal developmental trajectory from anovulatory to mature ovulatory cycles during adolescence remains poorly understood [64].

When natal families marry their daughters early, they preserve resources for other family members, which relate in particular to male children (who may reproduce within the household in the future) or adult sons and their wives. If the household is food insecure, offloading an adolescent daughter from the food budget may increase the odds of other children or grandchildren surviving. Therefore, early marriage of daughters may benefit the fitness of the remaining household members. However, the practice does not necessarily indicate conflict between daughters and parents, as early marriage might also transfer the daughter to a more food-secure household.

When marital households receive a young wife, they may benefit their own fitness both by accessing her subsistence effort, and also through her bearing children. Our analyses indicate that delayed pregnancy would likely benefit the woman’s lifetime fitness through enabling her to produce more robust offspring, and this has implications for the other household members given that the wife represents a ‘niche of maternal capital’ in which the husband’s genes (and hence those of his parents) are expressed during a period of development that has lifelong implications for health and fitness of the next generation. Our results show that whether early marriage is associated with poor nutritional status depends strongly on whether pregnancy is delayed or not, and only a minority of the population appear able to achieve this through access to contraception.

Overall, therefore, women’s nutritional status may be considered to be caught between the ‘survival’ interests of the natal family and the ‘reproductive’ interests of the marital household. Public health efforts could improve women’s nutritional status through efforts both to delay marriage and to delay pregnancy, but these efforts may need to be targeted in different ways at the natal and marital households.

### Strengths and limitations

Our analysis had both strengths and limitations. Ideally, we would analyse longitudinal data with women’s nutritional status measured at the time of both marriage and pregnancy. In practice, such data would be very difficult to obtain at scale in this setting. Our data derive from a cluster-randomized trial intended to improve maternal and infant nutritional status during pregnancy. It would be difficult to justify recruiting women into an observational study at the earlier time-point of marriage; moreover, adults in the marital household might be unwilling to provide consent for the wife to participate in such a study immediately after her marriage, as the majority of marriages in this population are illegal.

Regarding the data that were available, though only a subset of the full LBWSAT cohort, the sample was relatively large at both timepoints. There were missing data on both exposure and outcome, but we found negligible evidence that this introduced bias into our findings. Data at endpoint were obtained at varying times across a 25-month window; however, both BMI and MUAC displayed very minor and linear declines across this time period, allowing adjustment for time since delivery. Due to the large scale of the trial, anthropometry was undertaken by multiple data collectors, potentially introducing inter-observer error. However, our BMI findings were replicated by those for MUAC, whose measurement error is independent.

We lacked mechanistic data, on both physiological and behavioural traits, that could explain links of pregnancy or marriage age with BMI. We had no data on age at menarche or markers of biological fertility, we had only limited data on recent contraception use in a subsample and we could not address factors such as husband’s age or migration status, labour seasonality or other cultural norms that may affect reproductive/sexual behaviour.

We lacked a non-pregnant control group with repeat measurements over BMI, and so cannot be sure that the changes we detect through pregnancy relate directly to reproduction. However, BMI increases with age in this population [60] and we consider that short-term deficits in BMI of the magnitude we observed are highly likely to be due to the costs of lactation, as is widely seen in other populations [65]. Notably, the lack of longitudinal change in MUAC, contrasting with BMI, suggests that it may be less sensitive to short-term changes in individuals’ nutritional status. Finally, our data relate to a population where early marriage is the norm, and our findings may not apply to other populations with different norms of marital age.

## CONCLUSION

In a population where early women’s marriage and reproduction are the norm, we found that early pregnancy is the primary determinant of poor nutritional status. In contrast, the association of marriage age with nutritional status varied markedly by age at pregnancy. We found that earlier marriage is associated with poorer nutritional status of women, if they become pregnant immediately after marriage. This suggests that such women may be married to ameliorate nutritional stress of themselves and/or the remaining members of the natal household. Earlier marriage thus pushes the costs of promoting women’s nutritional status onto the marital household, where the variability now depends on the timing of pregnancy. We found that among those pregnant relatively late, earlier marriage was associated with better nutritional status, whereas for those becoming pregnant early, early marriage had limited the opportunity to accumulate ‘maternal capital’. Women’s nutritional status may thus be caught between the ‘survival’ interests of the natal family and the ‘reproductive’ interests of the marital household. Delaying pregnancy would benefit maternal and child health and potentially also fitness, as maternal undernutrition increases offspring mortality risk [66, 67]. However, the pressure for early reproduction in the marital household means that early pregnancy is common, further impairing nutritional status.

## SUPPLEMENTARY DATA

Supplementary data is available at *EMPH* online.

## Supplementary Material

eoac025_Supplementary_Data

## Data Availability

Requests to access the dataset for replicating the analyses, through a data sharing agreement, should be directed to Dr Naomi Saville, n.saville@ucl.ac.uk.
