# Supplementary material for: Associations of age at marriage and first pregnancy with maternal nutritional status in Nepal
Source: Evol Med Public Health. 2022 Jul 25;10(1):325–38. doi: 10.1093/emph/eoac025 (PMC9346504; doi:10.1093/emph/eoac025)
Supplement: eoac025_Supplementary_Data [file eoac025_supplementary_data.zip › Supplementary online material revised.pdf]

Evolution Medicine and Public Health: Supplementary online material

Scheduling of maternal capital: associations of age at marriage and first pregnancy with maternal nutritional status in Nepal

Jonathan CK Wells, Akanksha A Marphatia, Dharma S Manandhar, Mario Cortina-Borja, Alice M Reid and Naomi S. Saville

Figure S1

Study flowchart describing sample selection and data availability

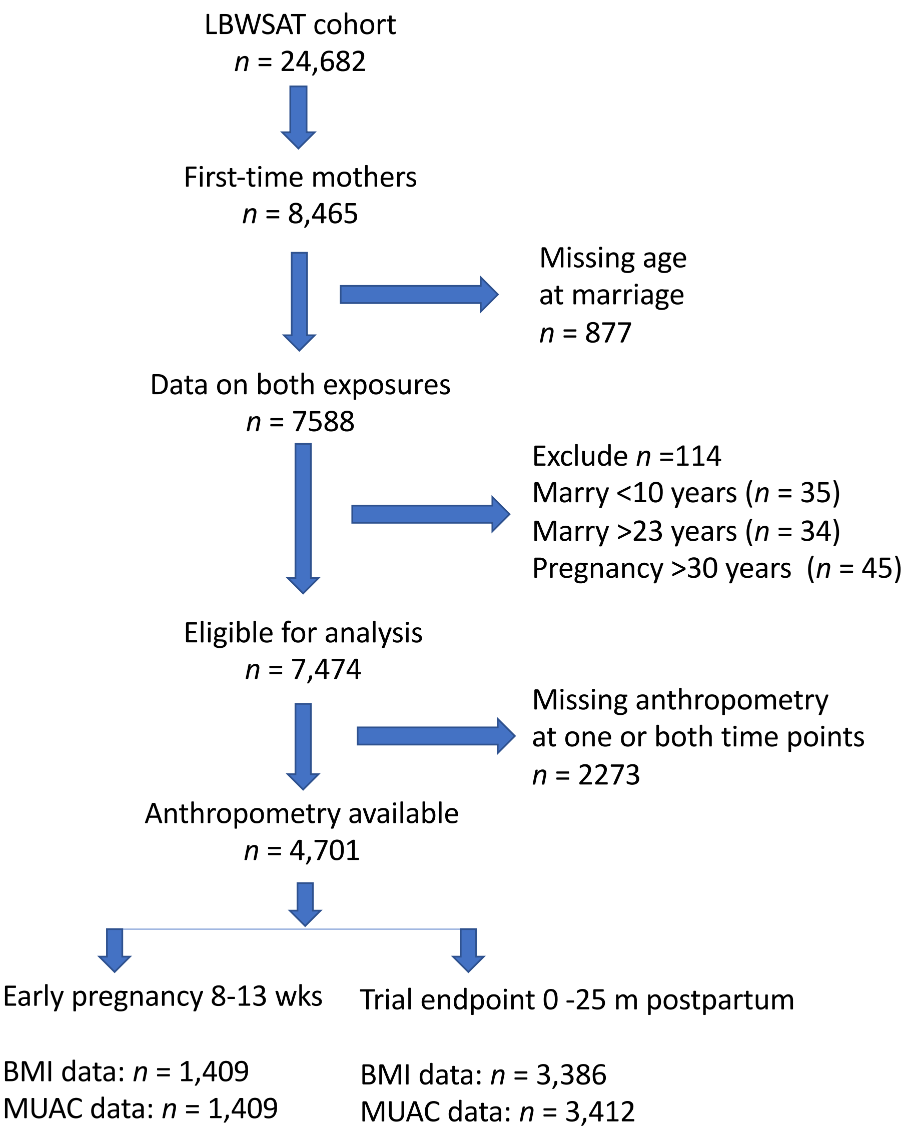

Table S1. Mutually adjusted associations of age at marriage and pregnancy with body mass index (BMI) and mid-upper arm circumference (MUAC) at endpoint

	Endpoint (*n* = 3,412)**					
	BMI (kg/m^2)			MUAC (cm)		
	B	95%CI	*p*-value	B	95%CI	*p*-value
Marriage age						
≤14 y	-0.74	-1.71, 0.22	0.13	-0.43	-1.31, 0.46	0.3
16 y	-0.33	-1.04, 0.37	0.3	-0.25	-0.90, 0.40	0.4
17 y	-0.22	-0.67, 0.22	0.3	-0.13	-0.53, 0.28	0.5
Pregnancy age						
≤15 y	-1.47	-2.07, -0.86	<0.001	-1.52	-2.08, -0.96	<0.001
16 y	-1.28	-1.85, -0.71	<0.001	-1.15	-1.68, -0.63	<0.001
17 y	-0.93	-1.40, -0.47	<0.001	-0.89	-1.32, -0.47	<0.001
18 y	-0.73	-1.06, -0.40	<0.001	-0.77	-1.08, -0.47	<0.001
Interaction term						
Marriage * Pregnancy age	-0.09	-0.16, -0.01	0.024	-0.07	-0.14, 0.00	0.054

* Mixed effects models, controlling for caste, maternal and paternal education, land ownership, assets, cluster, and study arm.

** 26 missing data points for BMI

Figure S2

The comparison of woman who were pregnant ≤15 years, stratified by their marriage age, was primarily of women who were pregnant at 15 years, stratified by marriage at 15 or 14 years. Therefore, in the majority of these women, assessment of nutritional status was made relatively soon after marriage.

Fig S2a: Among those pregnant ≤15 years, almost all women were pregnant at 15 years, whether they married at 15 years or ≤ 14 years.

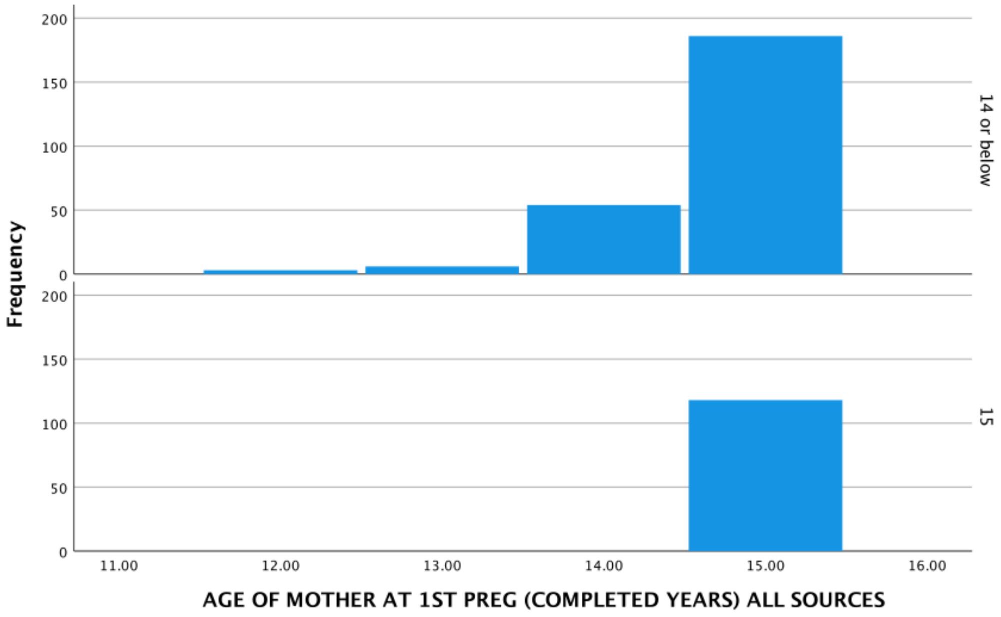

Fig S2b: Among those who married ≤ 14 years, most married at 14 years.

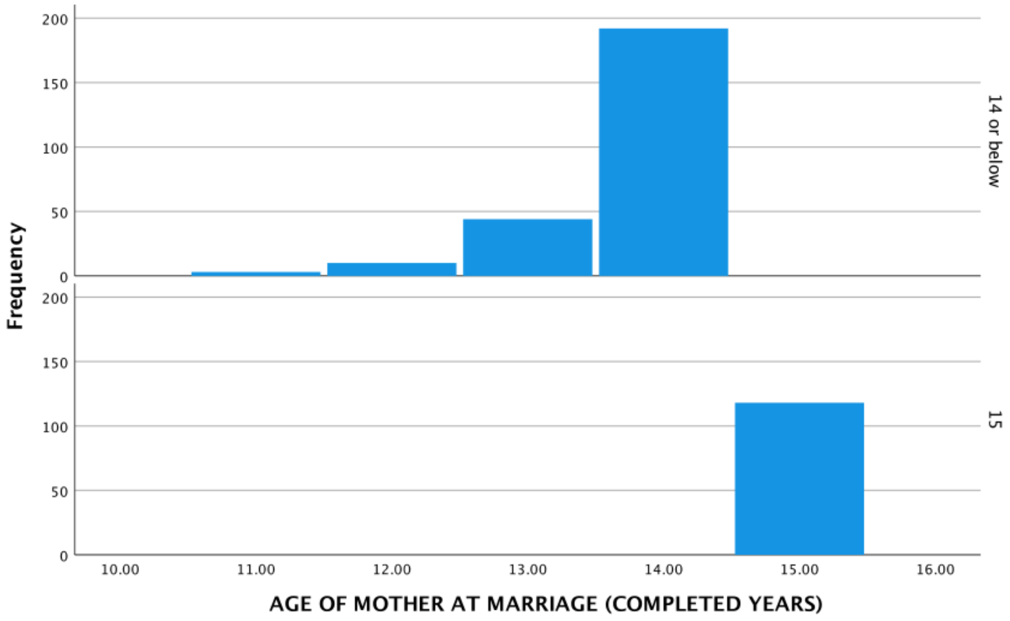

Figure S3.

Rates of women in a subsample (n = 2823) reporting contraception use in the month previous to the pregnancy, by age at marriage and age at pregnancy. For any marriage age, later pregnancy was associated with higher prevalence of contraception use, indicating that this practice contributed to later age at pregnancy. For women married early and pregnant at age 19+ years, contraception rates were low, indicating that after 18 years, the desire for children supersedes any effort to delay childbearing.

% reporting use of contraception

	**19+**	12.4	19.2	20.6	18.8
	**18**	26.3	19.6	15.1	14.4
**Pregnancy age**	**17**	20.0	17.5	15.0	20.3
	**16**	11.6	17.2	11.5	
	**<16**	9.8	11.4		
		**<15**	**15**	**16**	**17+**
			**Marriage age**		

Significant difference by Chi-square test, $p<0.0001$
